# Essential Gene Identification and Drug Target Prioritization in Aspergillus fumigatus

**DOI:** 10.1371/journal.ppat.0030024

**Published:** 2007-03-09

**Authors:** Wenqi Hu, Susan Sillaots, Sebastien Lemieux, John Davison, Sarah Kauffman, Anouk Breton, Annie Linteau, Chunlin Xin, Joel Bowman, Jeff Becker, Bo Jiang, Terry Roemer

**Affiliations:** 1 Merck Frosst Center of Fungal Genetics, Montreal, Quebec, Canada; 2 Department of Microbiology, University of Tennessee, Knoxville, Tennessee, United States of America; 3 Infectious Diseases, Merck Research Laboratories, Rahway, New Jersey, United States of America; Johns Hopkins University School of Medicine, United States of America

## Abstract

Aspergillus fumigatus is the most prevalent airborne filamentous fungal pathogen in humans, causing severe and often fatal invasive infections in immunocompromised patients. Currently available antifungal drugs to treat invasive aspergillosis have limited modes of action, and few are safe and effective. To identify and prioritize antifungal drug targets, we have developed a conditional promoter replacement (CPR) strategy using the nitrogen-regulated *A. fumigatus NiiA* promoter (p*NiiA*). The gene essentiality for 35 A. fumigatus genes was directly demonstrated by this p*NiiA*-CPR strategy from a set of 54 genes representing broad biological functions whose orthologs are confirmed to be essential for growth in Candida albicans and Saccharomyces cerevisiae. Extending this approach, we show that the *ERG11* gene family *(ERG11A* and *ERG11B)* is essential in A. fumigatus despite neither member being essential individually. In addition, we demonstrate the p*NiiA*-CPR strategy is suitable for in vivo phenotypic analyses, as a number of conditional mutants, including an *ERG11* double mutant *(erg11B*Δ, p*NiiA-ERG11A),* failed to establish a terminal infection in an immunocompromised mouse model of systemic aspergillosis. Collectively, the p*NiiA*-CPR strategy enables a rapid and reliable means to directly identify, phenotypically characterize, and facilitate target-based whole cell assays to screen A. fumigatus essential genes for cognate antifungal inhibitors.

## Author Summary

Aspergillus fumigatus is an opportunistic filamentous fungal pathogen of emerging clinical significance. Although virulence factors are seen as potential drug targets, neither genetic analyses nor genomic comparisons have identified genuine virulence factors in A. fumigatus. Essential genes required for fungal growth and viability also serve as potential drug targets, yet few have been described in this pathogen. To begin to catalog essential genes in *A. fumigatus,* we devised a genetic strategy for creating conditional mutants by promoter replacement of target genes using a nitrogen-regulated promoter. Applying this genetic approach to A. fumigatus genes orthologous to known essential genes of the nonpathogenic yeast, Saccharomyces cerevisiae and *Candida albicans,* we demonstrate a robust enrichment for identifying essential genes conserved within this pathogen. We show that A. fumigatus conditional mutants can be evaluated according to their terminal phenotypes (e.g., conidial germination, growth, morphology, and cidal versus static consequences) and pathogenesis in a murine model of systemic aspergillosis to prioritize essential genes as novel drug targets suitable for developing broad-spectrum antifungal agents.

## Introduction

Aspergillus fumigatus is a ubiquitous soil-dwelling saprophytic fungus that propagates through the prolific production of air-borne conidia [1]. Large-scale genome comparisons have shown that no gene sets are shared exclusively by both Aspergillus fumigatus and any other human pathogenic fungi sequenced to date, such as *Candida* or *Cryptococcus* species [2]. Thus, it has been recently suggested that A. fumigatus pathogenesis is based on its saprophytic lifestyle in combination with the immunosuppressed state of the host, rather than from genuine fungal virulence factors [2]. Although A. fumigatus conidia are constantly inhaled and seldom cause serious medical conditions in healthy individuals, immunocompromised patients (e.g., those with HIV infection or AIDS, solid organ and bone marrow transplant recipients, and those receiving chemotherapy) are highly susceptible to invasive aspergillosis, a fatal systemic infection [1,3,4]. Current treatment options for invasive aspergillosis are limited to three classes of antifungal therapeutics: polyenes (amphotericin B and various liposomal formulations), azoles (e.g., fluconazole, voriconazole, itraconazole), and, more recently, semisynthetic echinocandins (e.g., caspofungin and anidulafungin) [4,5]. Despite current therapeutic options, mortality associated with invasive aspergillosis remains high (ranging from 60% to 90%) and more efficacious antifungal drugs with novel mechanisms of action are needed [4,5].

The identification of conserved essential genes required for the growth of fungal pathogens offers an ideal strategy for elucidating novel antifungal drug targets. A comprehensive determination of all the essential genes has been achieved in the nonpathogenic yeast Saccharomyces cerevisiae [6,7]. Extending similar genetic approaches to fungal pathogens has proved difficult due to limited available molecular technologies and to the asexual nature of most medically relevant fungi, preventing the use of classical genetics. Nonetheless, large-scale essential gene identification in Candida albicans has begun through a number of alternative approaches, including antisense-based gene inactivation [8], transposon-based heterozygote screens for hypomorphs [9], homozygote null mutants [10], and a promoter replacement strategy to construct conditional mutants [11].

Large-scale functional analysis and essential gene identification in A. fumigatus have proved more difficult. Although gene disruption methodologies have been adapted to *A. fumigatus,* they are limited due to the organism's poor efficiency of homologous recombination as well as the inherent inability to study essential genes by such means. A. fumigatus essential genes have been defined using parasexual genetics in which gene essentiality is inferred from the failure to recover haploid segregants carrying a gene knock-out [12,13]. Nevertheless, such an approach is likely unsuitable to systematically identify all possible essential genes due to irregularities in the parasexual cycle of *A. fumigatus.* Direct demonstration of A. fumigatus gene essentiality and phenotypic analyses, however, may be achieved using molecular genetics strategies including RNA interference or promoter replacement strategies [14,15]. Mouyna et al. (2004) have used RNA interference to produce the predicted phenotypes associated with both a nonessential gene involved in melanin biosynthesis *(alb1)* and an essential glucan synthase, *FKS1* [14]. (Note: In this report, we maintain gene nomenclature for A. fumigatus genes as previously described [e.g., *alb1* or *FKS1*]; in cases where previously uncharacterized A. fumigatus genes are described, standard S. cerevisiae gene nomenclature and provisional gene names are adopted according to their yeast ortholog.) To date, conditional promoter replacement (CPR) strategies applied to A. fumigatus have been restricted to heterologous promoters including the *Aspergillus nidulans alcA* promoter and Escherichia coli tetracycline-regulated promoter [15,16].

Here we report a CPR strategy to identify A. fumigatus essential genes and to prioritize potential antifungal drug targets. This strategy uses the *A. fumigatus NiiA* nitrogen-regulatable promoter (p*NiiA*) to delete and replace the endogenous promoter of selected genes. By applying this strategy to 54 A. fumigatus genes of diverse biological functions whose orthologs are known to be essential for growth in S. cerevisiae and *C. albicans,* we have identified 35 genes essential for mycelial growth, with many displaying a cidal terminal phenotype. We also demonstrate that the *ERG11* gene family is essential in A. fumigatus and that the resulting p*NiiA*-CPR strains may be used as cell-based whole cell assays to examine target-specific chemical hypersensitivity. Finally, we show that a number of p*NiiA*-CPR mutants of essential genes fail to establish a terminal aspergillosis infection in a mouse model system. Therefore, both in vitro and in vivo phenotypic analysis of essential genes may be performed. This initial gene set comprises an experimentally validated drug target set that is broadly conserved within fungal pathogens and therefore of significant relevance to antifungal drug discovery.

## Results

### Construction of A. fumigatus Conditional Mutant by Promoter Replacement

**Figure 1:**
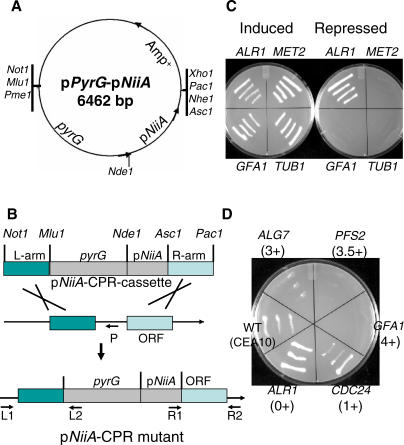
Outline of the A. fumigatus p*NiiA*-CPR Strategy (A) A cloning vector, p*PyrG*-p*NiiA,* was created specifically for the construction of CPR cassettes. This plasmid contains an *A. niger pyrG* (encoding orotidine-5′-phosphate decarboxylase and required for uridine-uracil prototrophy) as a selection marker [41], as well as an Amp^+^ selection marker and the A. fumigatus p*NiiA* conditional promoter. Unique restriction sites have been engineered at either side of the *pyrG-*p*NiiA* cassette to facilitate subcloning of flanking sequences. (B) Schematic overview of the p*NiiA*-CPR strategy and strain confirmation by PCR genotyping. Promoter replacement cassettes were constructed by inserting approximately 1.5 kb of homologous flanking sequences of the target gene (L-arm and R-arm) into the Not1/Mlu1 and Asc1/Pac1 restriction sites, respectively. After Not1 and Pac1 double digestion, the linearized p*NiiA*-CPR cassette was introduced into CEA17 strain *(pyrG^−^)* by protoplast transformation. As indicated, three sets of primers were used to perform genotypic PCRs to map the expected promoter replacement junctions (L1/L2 for left-arm junction, R1/R2 for right-arm junction) and to confirm the deletion of the native promoter (L1/P). (C) Phenotype of transformants obtained by the p*NiiA*-CPR strategy for *MET2, TUB1, GFA1,* and *ALR1.* CPR mutants were examined under p*NiiA*-inducing (AMM plus nitrate) and repressing (AMM plus ammonium) conditions after 36 h at 30 °C. (D) Growth phenotypes of *GFA1, PFS2, ALG7, CDC24,* and *ALR1* p*NiiA*-CPR mutants under these standard repressing conditions are scored qualitatively as the following: 4+, essential for cell viability, no growth under repressing conditions; 3.5+ or 3+, showing very strong or strong growth defect; 2+ or 1+, mild to minor growth defect; and 0+, no growth phenotype observed.

**Figure 2:**
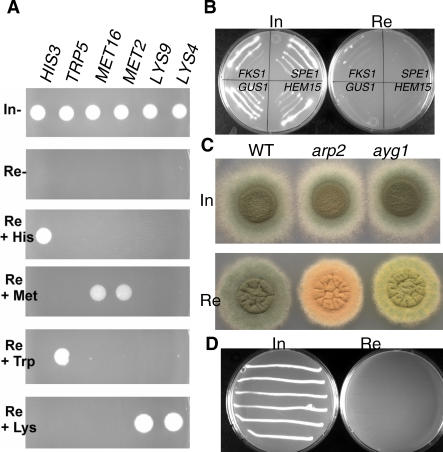
Genetic Evaluation of the p*NiiA*-CPR Strategy (A) Phenotypes of p*NiiA*-CPR mutants for *HIS3, TRP5, MET16, MET2, LYS9,* and *LYS4*. Under standard repressing conditions (Re, AMM plus ammonium), all strains lacked detectable growth (4+ phenotype). Growth was unimpaired under inducing conditions (In; AMM plus nitrate). Growth phenotypes of p*NiiA*-CPR mutants under repressing conditions were specifically suppressed if the cognate amino acid was provided to the growth media (His, histidine; Trp, tryptophan; Met, methionine; or Lys, lysine). (B) Growth phenotypes of p*NiiA*-CPR mutants for the previously reported A. fumigatus essential genes, *FKS1, GUS1, SPE2,* and *HEM15* [12,13]. Reproducible 4+ essential growth phenotypes are observed for each p*NiiA*-CPR mutant under repressing conditions with the exception of *FKS1,* which produced a 3.5+ growth phenotype. (C) *arp2* and *ayg1* conidial color phenotypes by the p*NiiA*-CPR strategy. Wild-type (WT) strain CEA10, p*NiiA*-*arp2,* and p*NiiA*-*ayg1* mutants display normal dark-green conidia color under inducing conditions. Gene-specific conidia color phenotypes characteristic of their known null phenotype [19] are specifically detected under repressing conditions. (D) *nudC* growth phenotype by the p*NiiA*-CPR strategy. Highly reproducible growth and morphological phenotypes associated with *nudC* mutants are observed (see Figure S1) as similarly determined using an *alcA* heterologous conditional promoter [15].

A. fumigatus conditional mutants were constructed by CPR using p*NiiA*. p*NiiA* was chosen based on previous work in both A. fumigatus and A. nidulans demonstrating its tight nitrogen-dependent transcriptional regulation [17,18]. Two operationally independent signals control p*NiiA* expression: an inducer (e.g., nitrate or other secondary nitrogen source) and a repressor (e.g., ammonium or other primary nitrogen source). Expression is achieved solely in the absence of ammonium and the presence of nitrate; however, repression is achieved by the presence of ammonium regardless of the presence of other nitrogen [17,18]. p*NiiA*-CPR mutants were constructed by homologous recombination-mediated deletion of the endogenous promoter (approximately 250 base pairs of promoter sequence immediately preceding the start codon of the gene) and replacement with an *NiiA* promoter cassette marked with the *pyrG* selectable marker (Figure 1A and 1B; see Materials and Methods for details). To test the p*NiiA*-CPR strategy, *TUB1* and *MET2* were first selected. *TUB1* encodes the broadly conserved and essential protein α-tubulin, and *MET2* encodes a homoserine *O*-acetyltransferase involved in methionine biosynthesis, mutations of which cause methionine auxotrophy. *TUB1* and *MET2* p*NiiA*-CPR mutants displayed wild-type mycelial growth on inducing medium (*Aspergillus* minimal medium [AMM] plus nitrate), but neither mutant was able to grow on repressing medium (AMM plus ammonium, Figure 1C). Furthermore, the *A. fumigatus MET2* conditional mutant displayed a tight methionine auxotrophy that was fully suppressed by methionine added to the medium (Figure 2A). p*NiiA*-CPR conditional mutants of *GFA1* and *ALR1,* whose orthologs are essential in both S. cerevisiae and *C. albicans,* were also examined (Figure 1C). *GFA1*, a glutamine-fructose-6-phosphate aminotransferase catalyzing the first step in the chitin biosynthesis pathway, was shown to be essential in *A. fumigatus.* However, CPR mutants of *ALR1* (a metal cation transporter belonging to a gene family in A. fumigatus) failed to display any growth defect under repressing conditions, revealing that *ALR1* is nonessential in A. fumigatus (see below).

According to the severity of the growth phenotype displayed by CPR mutants in repressing conditions, qualitative scores were assigned to each of the CPR mutants to classify the terminal phenotype (Figure 1D). A 4+ shutoff phenotype was assigned to those strains (e.g., *GFA1*) that completely fail to grow on repressing medium after 48 h at 30 °C. Similarly, a 3.5+ or 3+ shutoff phenotype was used to score those strains that showed a very severe (e.g., *PFS2*) or a severe (e.g., *ALG7*) growth phenotype, respectively. Other growth defects were scored as 2+ (mild), 1+ (minor; e.g., *CDC24*), or 0+ (no defect; e.g., *ALR1*). All essential genes described here showed either a complete absence of growth (4+) or a dramatic growth defect (3.5+ and 3+) under repressing conditions.

### Evaluating p*NiiA*-CPR Reliability by Genetic Approaches

To test its reliability, the p*NiiA*-CPR strategy was further applied to multiple A. fumigatus genes whose null phenotype was either known or independently verified by other means. First, p*NiiA*-CPR mutants were constructed for five genes *(HIS3, TRP5, MET16, LYS9,* and *LYS4)* involved in amino acid biosynthesis. Growth was completely impaired for all strains under repressing conditions lacking amino acids but fully restored when the cognate amino acid was provided (Figure 2A). Second, p*NiiA*-CPR mutants were constructed for four genes *(FKS1, GUS1, SPE2,* and *HEM1)* whose essentiality in A. fumigatus has been demonstrated by a parasexual genetic strategy [12,13]. In each instance, the conditional mutant displayed an essential growth phenotype (Figure 2B). Third, we constructed p*NiiA*-CPR mutants of two nonessential genes *(ayg1* and *arp2)* involved in conidial pigmentation whose null phenotypes result in the production of yellow-green and pink-red conidiospores, respectively [19]. Hence, conidial pigmentation phenotypes of *ayg1* and *arp2* mutants serve as whole cell reporters to monitor the level of repression achieved by the p*NiiA*-CPR system. Indeed, p*NiiA*-*ayg1* and p*NiiA*-*arp2* conidia color shifted from dark-green (wild-type conidia color) under inducing conditions to yellow-green *(ayg1)* or red-pink *(arp2)* under repressing conditions (Figure 2C). Finally, we applied this p*NiiA*-CPR strategy to *A. fumigatus nudC,* a gene shown to be essential by promoter replacement with *A. nidulans alcA* promoter [15]. Consistent with a previous report [15], the p*NiiA*-*nudC* mutant displayed (1) an essential growth phenotype (Figure 2D), (2) a germination defect of conidia with approximately 70% of conidia failing to germinate and approximately 30% of conidia forming short growth-arrested germ tubes (Figure S1), and (3) an *nudC* nuclear distribution terminal phenotype characterized by multinucleate conidia (Figure S1).

An advantage to the use of large homologous flanking sequences to p*NiiA*-CPR cassettes is that multiple independent promoter replacement mutants could be recovered for a given gene. Thus, gene essentiality can be independently validated from multiple p*NiiA*-CPR mutants. Indeed, multiple independent p*NiiA*-CPR mutants (*n* > 2) were recovered for 18 of the 19 genes mentioned above, and in all cases, their independent p*NiiA*-CPR mutants displayed reproducible growth phenotypes under repressing conditions. Although the essential growth phenotype of *GUS1* was based on the single p*NiiA*-*GUS1* mutant recovered, its essentiality is consistent with previous work and with its predicted function as a glutamate-tRNA sythetase [13].

### Direct Comparison between p*NiiA*-CPR and Gene Disruption Terminal Phenotypes

Reliability of the p*NiiA*-CPR system was also assessed by *pyrG*-based gene disruption of multiple genes shown to be essential by the p*NiiA*-CPR approach (Table 1). In fact, viable null mutants of *ERG11A, ERG11B, ERG27, ROM2,* and *MET2* (when exogenous methionine was provided) were recovered, consistent with their nonessential or conditional essential phenotypes determined by p*NiiA*-CPR mutants. Moreover, no viable null mutants were recovered for either *TOM40* or *LUC7* by gene disruption (two essential genes identified by the p*NiiA*-CPR approach) despite a significantly large number of transformants examined (84 for *TOM40* and 107 for *LUC7,* respectively) (Table 1). Thus, the essentiality of *TOM40* and *LUC7* is shown by both the p*NiiA*-CPR strategy and gene deletion analysis. Collectively, these data demonstrate that robust genetic conclusions spanning an extensive set of “phenotypic reporter genes” are reliably reproduced by the p*NiiA*-CPR strategy.

**Table 1:**
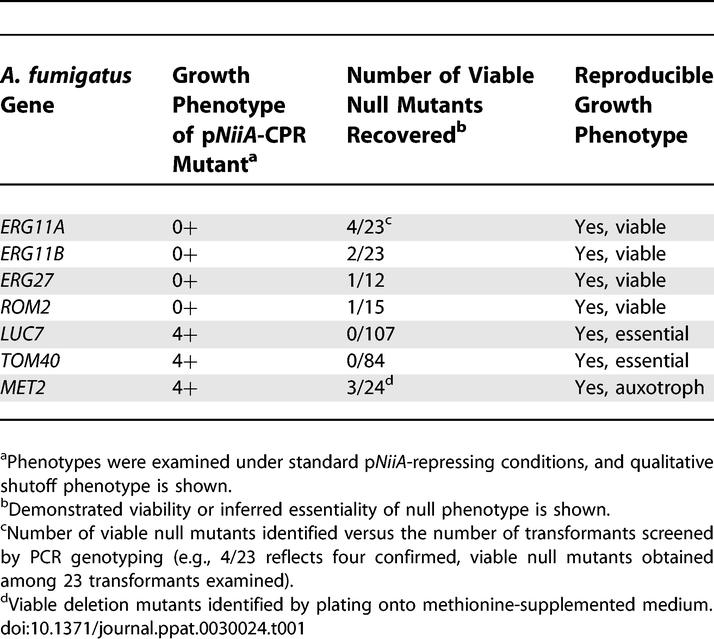
Comparison of Growth Phenotypes Observed by p*NiiA*-CPR Methods versus Gene Knockout

### Quantitative Analysis of p*NiiA*-CPR Transcriptional Regulation

**Figure 3:**
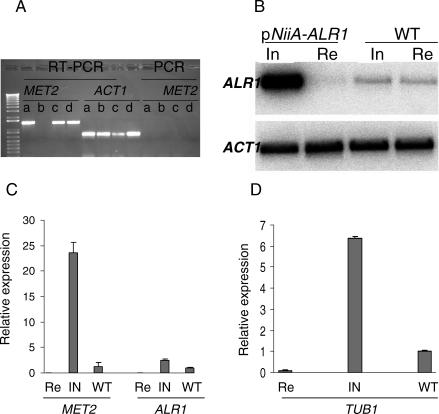
Expressional Level Analysis of p*NiiA*-CPR Mutants (A) RT-PCR of p*NiiA-MET2:* (a) p*NiiA*-*MET2* mutant under inducing conditions, (b) p*NiiA*-*MET2* mutant under repressing conditions plus 100 μg/ml methionine, (c) p*NiiA*-*MET2* mutant under inducing condition plus 100 μg/ml methionine, and (d) wild-type A. fumigatus strain (CEA10) under repressing conditions. To monitor and ensure even sample loading, RT-PCRs for the *ACT1* transcript were also performed using identical samples. In addition, standard PCR was performed to confirm that there is no detectable genomic-DNA contamination. (B) Northern blot analysis of p*NiiA*-*ALR1* mutant expression levels. Northern blot was performed with RNA samples prepared from the nonessential *ALR1* mutant and wild-type cells growing in standard inducing (In) or repressing (Re) conditions. *ACT1* transcript levels served as sample loading controls. (C) Real-time RT-PCR analysis of expression level of p*NiiA*-*ALR1* and p*NiiA*-*MET2*. p*NiiA*-CPR mutants and wild-type strain (CEA10) were grown in inducing (In) or repressing (Re) medium at 37 °C for 20 h, and total RNA was extracted from identical time points. The relative expression level normalized to total input RNA [43] is displayed on the *y*-axis. Error bars represent SD. Compared to wild-type, the relative expression level for *ALR1* and *MET2* is 0.013 and 0.061 under repressing conditions and 2.49 and 23.58 under inducing conditions, respectively. (D) *pNiiA-TUB1* expression level under inducing (In) and repressing (Re) conditions versus wild-type level is displayed on the *y*-axis. Error bar represents SD. The relative expression level of p*NiiA-TUB1* versus wild-type is 0.06 under repressing conditions and 6.35 under inducing conditions, respectively. Note: Since real-time RT-PCR was performed using primers detecting both p*NiiA-TUB1* and wild-type allele, data shown were calculated by subtracting wild-type level from total inducing (In) and total repressing (Re) level, respectively.

Reverse transcription (RT)-PCR was first performed to monitor p*NiiA*-CPR gene expression levels for all previously described genes in Table 1. As p*NiiA*-*MET2* mutants grow normally in repressing media supplemented with methionine, *MET2* mRNA expression could be assessed in both inducing and p*NiiA*-repressing conditions by RT-PCR. *MET2* mRNA was detected in both the p*NiiA*-*MET2* mutant and wild-type CEA10 on inducing medium; however, no *MET2* transcript was detected in the p*NiiA*-*MET2* mutant on repressing medium supplemented with methionine (Figure 3A). Similarly, mRNA transcripts were detected by RT-PCR in CEA10 and p*NiiA*-CPR mutants for *ERG11A, ERG11B, ERG27, ALR1,* and *ROM2* under inducing conditions but not from p*NiiA*-CPR mutants maintained under repressing conditions (Figure S2). In addition, Northern blot analysis also confirmed a significant depletion of *ALR1* mRNA levels under repressing conditions (despite the lack of a clear growth phenotype) versus its endogenous level of expression in wild-type CEA10 (Figure 3B). Although the *ALR1* mRNA levels of the *ALR1* conditional mutant were clearly elevated compared to the wild-type, no deleterious growth phenotype was detected (Figure 1C).

Real-time RT-PCR was performed to further evaluate the achievable range of expression levels for three p*NiiA*-CPR mutants shown to yield either a nonessential *(ALR1),* a conditional essential *(MET2),* or an essential *(TUB1)* terminal phenotype (Figure 3C and 3D). Compared to wild-type, *ALR1* and *MET2* expression levels in CPR mutants were dramatically reduced by 75.9-fold and 16.3-fold under repressing conditions and elevated by 2.8-fold and 23.6-fold under inducing conditions, respectively (Figure 3C). To overcome the technical difficulty of performing real-time RT-PCR on an essential gene, a *TUB1* tandem duplication mutant was constructed in which the p*NiiA*-*TUB1* allele is balanced by a wild-type *TUB1* copy under the control of its native promoter. Thus, expression of the p*NiiA*-regulated *TUB1* allele could be monitored under repressing conditions (see Materials and Methods). Real-time RT-PCR performed with this strain revealed expression of the p*NiiA*-*TUB1* allele to be approximately 0.06-fold when repressed and 6.3-fold under inducing conditions relative to normal *TUB1* expression levels (Figure 3D). Collectively, these data demonstrate that the p*NiiA*-CPR strategy typically achieves tight regulatable expression and suggest that it could be reliably applied to large-scale analysis of gene essentiality in A. fumigatus.

### Large-Scale Identification of Essential Genes

The genomic sequence of A. fumigatus strain AF293 [20], as well as a second clinical isolate used in this study, CEA10, where approximately 10× coverage has been obtained (N. Fedorova, V. Joardar, J. Crabtree, M. Anderson, R. Maiti, et al., unpublished data), provides an opportunity to systematically identify essential genes. Although global annotation of the A. fumigatus genome is ongoing, homologs to genes of interest were identified through search alignment tools and corresponding promoter replacement cassettes designed accordingly. In total, 54 A. fumigatus genes were selected for p*NiiA*-CPR analysis, of which 49 are known to be essential (or conditionally essential) for growth in S. cerevisiae with all but three of these being essential for normal growth in C. albicans (Table 2). In addition, genes were selected so as to represent a broad diversity of gene functions and cellular processes. Biasing this A. fumigatus gene set toward those essential in both S. cerevisiae and C. albicans builds on previous work demonstrating that at least 60% of C. albicans genes homologous with essential S. cerevisiae genes were also essential in C. albicans [11]. Therefore, we reasoned that a set of genes experimentally demonstrated as essential in two distinct hemiascomycetes would be highly enriched for genes sharing conserved essential functions within the euascomycetes.

**Table 2:**
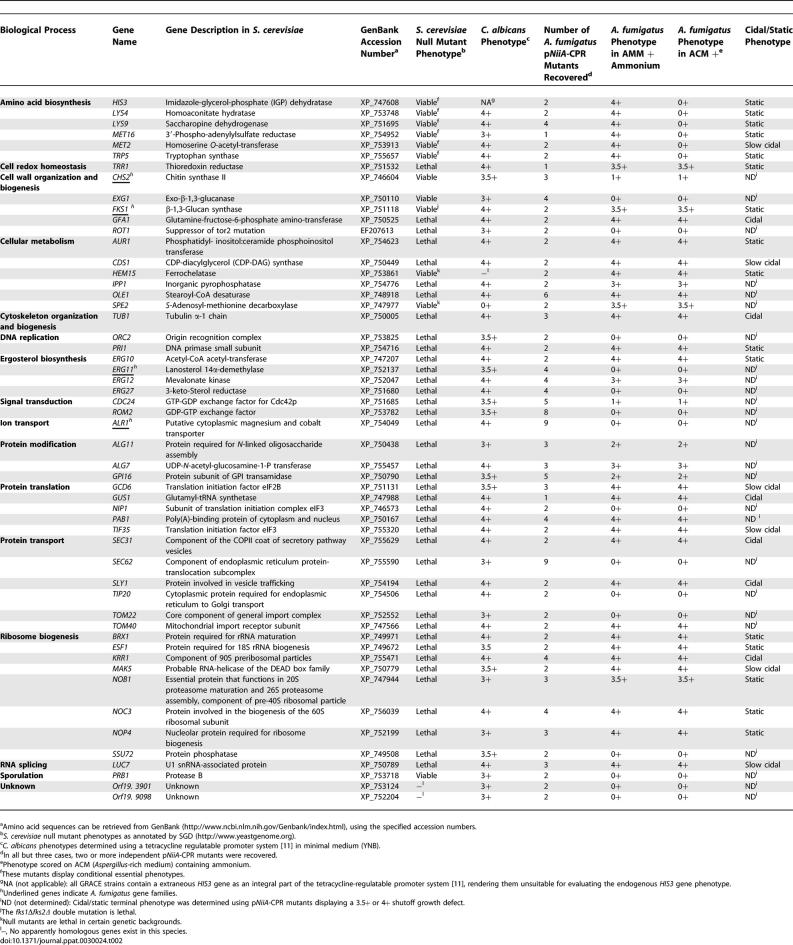
Growth Phenotypes of A. fumigatus Gene Set Examined and Concordance of Gene Essentiality in C. albicans and S. cerevisiae

Analysis of this A. fumigatus gene set revealed 35 genes displaying a 3+ or greater (3.5+ or 4+) essential growth phenotype (Table 2). Excluding genes involved in amino acid biosynthesis, no significant differences in terminal growth phenotypes were observed between minimum medium (AMM) and rich medium (*Aspergillus* complete medium [ACM]). Essential genes comprise a spectrum of biological functions including lipid, ergosterol, cell wall, amino acid, protein, and heme biosynthesis, as well as glycosylation, secretion, RNA processing, and novel genes of unknown functions (Table 2). Moreover, among those genes displaying an essential phenotype in both S. cerevisiae and C. albicans and predicted to have only a single ortholog in A. fumigatus (*n* = 44), 32 genes (73%) are experimentally demonstrated as sharing a conserved growth phenotype of 3+ or greater in *A. fumigatus.*

**Figure 4:**
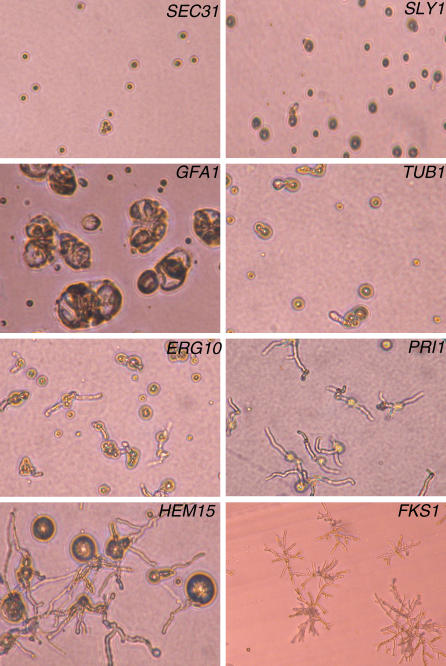
Analysis of p*NiiA*-CPR Associated Morphological Terminal Phenotypes Terminal growth phenotypes of p*NiiA*-CPR mutants were observed under a microscope (×160) with conidia grown for 36 to 40 h at 30 °C under standard repressing conditions. A continuum of conidia germination phenotypes of high penetrance was observed; ranging from those completely failing to undergo polarized growth *(SEC31, SLY1)* or swollen and highly disorganized condidia *(GFA1),* to those displaying stunted *(TUB1, ERG10)* or nonbranching germlings with swollen conidia *(HEM15)* with only rudimentary polarized growth. Micromycelial colonies were observed for a p*NiiA*-*FKS1* mutant and resembling the morphology of wild-type A. fumigatus when grown in the presence of minimum effective concentration (MEC) of the FKS1p inhibitor, caspofungin [21]. Growth phenotypes under inducing conditions are shown in Figure S3.

Essential genes identified in this manner constitute a collection of possible antifungal drug targets, and microscopic examination of their terminal growth phenotypes may assist in their prioritization (Figure 4). For example, repression of *GFA1* produced enlarged and highly disrupted nongerminating conidia that failed to undergo any polarized growth. As *GFA1* is predicted to encode the sole glutamine-fructose-6-phosphate aminotransferase activity responsible for the first and rate-limiting step in chitin synthesis, this terminal phenotype likely reflects the normal interdependence between cell wall biosynthesis and polarized growth. Conidia of *SEC31* and *SLY1* incubated under repressing conditions were unable to germinate, suggesting that these genes are required for the initiation of germ tubes, whereas terminal phenotypes of *TUB1, ERG10,* and, most prominently*, PRI1,* resulted in growth-arrested germ tubes. Interesting, *HEM15* displayed a more complex terminal phenotype of dramatically swollen conidia with single or multiple germ tubes. Repression of p*NiiA*-*FKS1* yielded stubby and highly branched micromycelial germlings that morphologically resemble the chemotype observed when A. fumigatus is treated with Fks1p-specific inhibitors such as caspofungin and related echinocandins [21]. Thus, distinct terminal phenotypes are identified among p*NiiA*-CPR mutants, and in some instances, they appear more severe than that caused by either genetic or chemical inactivation of *FKS1*.

**Figure 5:**
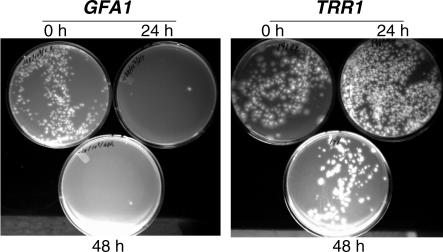
Determination of Cidal or Static Terminal Phenotypes A representative example of cidal and static terminal phenotypes for *GFA1* and *TRR1* p*NiiA*-CPR mutants is shown. (A) p*NiiA*-GFA1 displayed a cidal terminal phenotype as a dramatic (greater than 90%) reduction in CFU was observed after incubation in p*NiiA*-repressing conditions for 24 or 48 h. (B) p*NiiA*-*TRR1* revealed a static terminal phenotype as no significant reduction in CFU counts was detected after 48-h incubation under repressing conditions. A summary of all additional cidal/static terminal phenotypes for A. fumigatus genes displaying 4+ qualitative growth phenotypes is provided (see Table 2).

Essential genes with a cidal terminal phenotype are commonly preferred since genetic evidence predicts that chemical inhibitors of such targets may display similar chemotypes. An assay to evaluate cidal or static terminal phenotypes was applied to essential genes displaying a 3.5+ or 4+ growth phenotype (Table 2) by incubating conidia in p*NiiA*-repressing medium for various durations of time before washing and plating conidia onto p*NiiA*-inducing medium where viability was scored by counting colony-forming units (CFU) (see Materials and Methods and Figure 5). Of 28 genes tested, six genes (approximately 21%), including *GFA1* (Figure 5), displayed a cidal terminal phenotype, as scored by a significant reduction (greater than 90%) of CFU after 24-h incubation in repressing medium. Additional genes demonstrating a cidal terminal phenotype include *KRR1, SEC31, SLY1, TUB1,* and *GUS1* (Table 2). Additional genes displayed a slow cidal phenotype, as scored by significant reduction (greater than 90%) in CFU after 48- or 72-h incubation in repressing medium, or a static terminal phenotype after 48-h incubation in repressing conditions as shown with *TRR1* (Figure 5, Table 2).

### The *ERG11* Gene Family Is Essential in A. fumigatus

**Figure 6:**
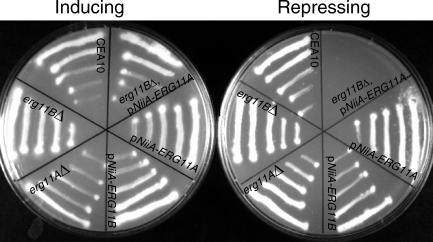
Phenotypic Analyses of *ERG11* Gene Family Growth phenotypes of *ERG11* gene family were observed with p*NiiA*-CPR mutants, null deletion mutants, and double mutants *(erg11B*Δ, p*NiiA-ERG11A).* Strains were grown on either inducing medium (AMM plus nitrate) or repressing medium (AMM plus ammonium) at 30 °C for 40 h.

Identifying gene families that are essential for the growth and viability of A. fumigatus can be missed by our approach and thus require additional considerations. For example, duplicate *ERG11* genes (encoding 14α-demethylase, the known target of azole-based antifungal agents) exist in A. fumigatus [22,23]. While disruption of *ERG11A* displays no effect on growth or ergosterol levels [23], it remained unknown whether *ERG11B* alone or the gene pair together is essential in A. fumigatus. To address this, individual p*NiiA*-CPR mutants of *ERG11A* and *ERG11B* were constructed, with neither producing a noticeable growth defect under repressing conditions (Figure 6, Table 1). Further, viable *erg11A*Δ and *erg11B*Δ null mutants displaying robust growth were readily recovered (Figure 6, Table 1). Thus, these data demonstrate that neither member of the *ERG11* gene family is essential individually in A. fumigatus. To examine whether the *ERG11* gene family is essential, a double mutant was constructed by creating a p*NiiA*-*ERG11A* allele within an *ERG11B* null mutant background (see Materials and Methods). The resulting *ERG11* double mutant *(erg11B*Δ, p*NiiA*-*ERG11A)* displays robust growth under the inducing condition, with no growth evident under repressing conditions (Figure 6). Therefore, these data suggest that either of the *ERG11* gene pair can functionally compensate for loss of the other, and the genetic inactivation of both *ERG11A* and *ERG11B* indicates their essentiality in A. fumigatus.

### In Vivo Analysis of p*NiiA*-CPR Mutants in a Murine Model of Systemic Aspergillosis

**Figure 7:**
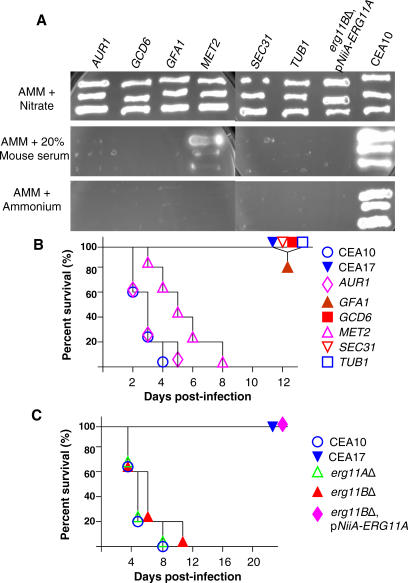
In Vivo Validation of A. fumigatus p*NiiA*-CPR Mutants in an Immunocompromised Murine Model of Systemic Infection (A) p*NiiA*-CPR mutants growing on AMM plus 20% mouse serum (Sigma) reproduced their terminal growth phenotypes as observed on either AMM plus ammonium or rich medium (Table 2). (B and C) In vivo validation of p*NiiA*-CPR mutants. (B) ICR male mice were immunocompromised by administration of cyclophosphamide at 150 mg/kg twice prior to infection and 100 mg/kg twice a week after infection. Approximately 10^5^ viable conidia from individual p*NiiA*-CPR mutants *(TUB1, SEC31, GCD6, GFA1, MET2,* and *AUR1)* were injected into the tail vein of immunocompromised mice (five mice per group). Signs of infection were monitored for up to 12 d following infection. Wild-type strain CEA10 and the starting strain CEA17 (a *pyrG* ^−^ auxotroph of CEA10) [25,39] were included as positive controls for virulence and avirulence, respectively. (C) Genetic inactivation of the *ERG11* gene family promotes avirulence in an immunocompromised murine model of systemic infection. Pathogenesis of *erg11A*Δ, *erg11B*Δ, and an *ERG11* double mutant *(erg11B*Δ, p*NiiA-ERG11A)* was similarly analyzed (as described above) but over a longer postinfection period (22 d), and animal survival was compared to CEA10 and CEA17 control strains.

Animal models of fungal pathogenesis provide important experimental verification whether genetic mutations abrogate (or attenuate) the virulence of the pathogen. As mouse serum contains approximately 200 μM ammonium [24] and such levels of ammonium are theoretically sufficient to repress the p*NiiA* promoter, we tested this by streaking p*NiiA*-CPR mutants onto AMM plates containing 20% mouse serum (Figure 7A). In each case, *TUB1, SEC31, GCD6, GFA1, MET2, AUR1,* and p*NiiA*-*ERG11A, erg11B*Δ CPR mutants reproduced their terminal growth phenotypes as observed on either AMM or rich medium (Table 2), demonstrating that mouse serum levels of ammonium (or other primary nitrogen source) are sufficient to repress the *NiiA* promoter. Indeed, as even higher ammonium levels (approximately 1 to 4 mM) of repressor exist in mouse tissues (e.g., liver, kidney, brain, muscle) [24], we reasoned that the virulence of p*NiiA*-CPR mutants could be examined in a murine model of aspergillosis. To test this, an animal model was first established using mice that were immunocompromised by cyclophosphamide and infected with 100,000 conidia by tail vein injection (see Materials and Methods). Control experiments demonstrate that all mice (*n* = 5) infected with the wild-type virulent strain, CEA10, died from infection within 8 d, while 100% survival was observed for mice (*n* = 5) infected with a known avirulent strain, CEA17 [25]. Virulence of CPR mutants of *TUB1, SEC31, GCD6, GFA1, AUR1,* and *MET2* was similarly examined (Figure 7B). No mortality or morbidity was observed for mice infected with p*NiiA*-*TUB1* over the 12-d period, as predicted for a core essential gene that is significantly repressed and for which sufficient in vivo ammonium levels exist. Similarly, p*NiiA*-based repression was sufficient to fully abrogate virulence of *SEC31, GFA1,* and *GCD6* (Figure 7B). Further, whereas both *erg11A*Δ and *erg11B*Δ mutants were similarly virulent as the wild-type CEA10, an *ERG11* double mutant *(erg11B*Δ, p*NiiA*-*ERG11A)* was fully avirulent over an extended 22-d period (Figure 7C). On the other hand, attempted repression of *MET2* and *AUR1* had no effect on A. fumigatus virulence (Figure 7B). As p*NiiA*-*MET2* growth was unaffected by serum, and a *C. albicans MET2* tetracycline-regulatable conditional mutant is similarly virulent [10], this likely reflects that sufficient levels of methionine are scavenged to support growth both in vitro and in vivo. Although the observed virulence phenotype of p*NiiA*-*AUR1* remains unclear (see Discussion), we demonstrate that p*NiiA*-CPR mutants generally gave in vivo phenotypes consistent with reliable repression being achieved.

### Chemical Hypersensitivity of p*NiiA*-CPR Mutants

In principle, partially depleting the activity of a particular gene product sensitizes cells to compounds that act through that target. Modulating gene dosage may be achieved by deleting one copy in a diploid organism [6,26], antisense interference [8], or reduced gene expression through titratable repression of a regulatable promoter [11]. To test whether intermediate expression levels of p*NiiA*-CPR mutants could be achieved to produce chemical hypersensitivity, we first examined the chemical sensitivity of a p*NiiA*-*ALG7* mutant to its cognate inhibitor, tunicamycin. A partial depletion of Alg7p was inferred by growing p*NiiA*-*ALG7* in the presence of an ammonium/nitrate ratio sufficient to reduce growth rate by approximately 50%. Under such conditions, depletion of Alg7p resulted in an 8- to 16-fold shift in minimum inhibitory concentrations (MICs) to tunicamycin without any significant change in sensitivity to either fluconazole or itraconazole (Table 3). Moreover, no significant change in tunicamycin MIC was observed across a panel of similarly sensitized p*NiiA*-CPR mutants (Table 3). Therefore, partial depletion of the A. fumigatus Alg7p resulted in a specific hypersensitivity to tunicamycin.

**Table 3:**
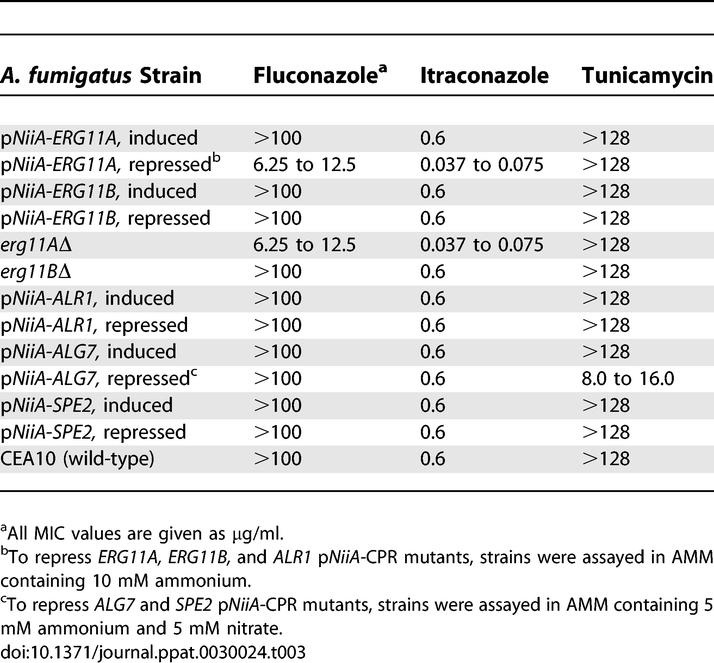
MIC of Antifungal Drugs to p*NiiA*-CPR Strains

A. fumigatus exhibits an extremely low susceptibility to fluconazole (MIC > 256 μg/ml), a feature attributed to the genetic redundancy of *ERG11* in this pathogen [22,23]. Using the conditional mutants, we tested whether inactivation of either member of the *ERG11* gene family may sensitize A. fumigatus to *ERG11*-specific inhibitors (Table 3). As neither *ERG11* gene displays growth defects under full repressing conditions, p*NiiA*-CPR mutants of *ERG11A* and *ERG11B* serve as whole cell assays by growing each mutant under full repressing conditions and determining their respective MIC to fluconazole. Indeed, the p*NiiA*-*ERG11A* mutant displayed an 8- to 16-fold lower MIC to fluconazole; no detectable drug hypersensitivity was observed for either p*NiiA*-*ERG11B* or control strains tested (Table 3). MIC determinations using null mutants of *erg11A*Δ and *erg11B*Δ independently confirmed that depletion of *ERG11A* (rather than *ERG11B*) similarly sensitizes A. fumigatus 8- to 16-fold to fluconazole. Moreover, only depletion of *ERG11A,* as either a p*NiiA*-CPR mutant or null mutant, produced hypersensitivity to itraconazole, another triazole that, unlike fluconazole, displays strong activity against wild-type A. fumigatus (Table 3). Neither p*NiiA*-CPR mutants nor null mutants of *ERG11A* or *ERG11B* displayed a change in MIC to tunicamycin. This preferential drug sensitivity of mutants depleted of *A. fumigatus ERG11A* further supports the feasibility of target-based sensitized whole cell assays using p*NiiA*-CPR mutants*.* These data also suggest *ERG11A* likely encodes the major 14α-demethylase activity and that *ERG11A,* rather than *ERG11B,* largely contributes to the poor susceptibility of A. fumigatus to fluconazole (see Discussion).

## Discussion

In this report, we describe a p*NiiA*-CPR strategy to construct A. fumigatus conditional mutants and to directly identify and evaluate gene essentiality in vitro and in vivo. Replacement of the endogenous gene promoter with p*NiiA* typically enables a robust shutoff of gene expression to assess terminal growth phenotypes including intracellular, morphological, and cidal growth consequences. Collectively, the completion of the A. fumigatus genomic sequence, an understanding of essential genes in other yeasts, and the p*NiiA*-CPR strategy enable a rational approach to determine the essentiality of orthologous genes in A. fumigatus. Indeed, genomic comparisons between A. fumigatus and other eukaryotes reveals that, in all but one of 131 largely essential eukaryote orthologous groups, an A. fumigatus ortholog is identified [27]. Here we demonstrate that at least 73% of the predicted essential genes we examined were essential for growth. Hence, a broad spectrum of antifungal targets conserved within major human fungal pathogens was efficiently identified. We also demonstrate how this approach can be extended to gene families (e.g., *ERG11*) and that the resulting p*NiiA*-CPR mutants serve as target-based whole cell assays suitable for drug screening.

To evaluate the utility of the *NiiA* conditional promoter for studying gene essentiality, an extensive analysis of its regulatable expression was performed. First, its reliability was examined by genetic means. Conditional mutants of multiple genes encoding functions involved in amino acid biosynthesis were constructed, and all showed 4+ shutoff growth phenotypes that could be reversed by adding back appropriate amino acids to the medium (Figure 2A, Table 2). In addition, A. fumigatus genes examined by the p*NiiA*-CPR strategy and independently examined by a parasexual genetics strategy [12,13] yielded reproducible essential growth phenotypes (Figure 2B). p*NiiA*-CPR mutants of genes in the melanin biosynthetic pathway also gave the predicted terminal conidia color change characteristic to their known null phenotype (Figure 2C) [19]. Thus, genetic data strongly suggest that sufficiently achievable repression levels are obtained by the p*NiiA* promoter independent of the gene to which it is linked or its physical location within the genome. We also directly compared terminal phenotypes of *A. fumigatus nudC* genes achieved by p*NiiA*-CPR strategy to that achieved by the *alcA* conditional promoter system [15] and obtained highly comparable *nudC* terminal phenotypes resembling its reported null phenotype (Figures 2D and S1). Finally, a comparative analysis was performed of terminal phenotypes for seven genes evaluated by both p*NiiA*-CPR and gene disruption strategies. By both methods, three genes *(LUC7, TOM40,* and *MET2)* were shown to be essential, while four genes *(ERG11A, ERG11B, ERG27,* and *ROM2)* were found to be nonessential (Table 1).

Genetic interrogation of the p*NiiA*-CPR strategy supports a general reliability of this method, but quantitative metrics for expression levels achieved by the *NiiA* promoter were also required. Multiple genes monitored by RT-PCR displayed repression levels noticeably below wild-type expression levels (Figure 3A; also see Figure S2). Further, real-time RT-PCR analysis of expression levels among p*NiiA*-CPR mutants for *ALR1*, *MET2*, and *TUB1* demonstrates achievable repression levels in the range of approximately 16- to 76-fold below wild-type (Figure 3C and 3D). In several cases, overexpression was detected by RT-PCR and/or real-time RT-PCR under inducing conditions. Although no deleterious growth consequences were observed, intrinsic overexpression may complicate p*NiiA*-CPR mutant construction and/or analysis in some instances.

Despite a high concordance in A. fumigatus gene essentiality to known essential S. cerevisiae and C. albicans orthologs*,* several exceptions were observed. In some instances, this is due to gene redundancy, as we have demonstrated by our genetic analysis of the *ERG11* gene family and may similarly relate to *ALR1*. However, other nonessential phenotypes determined for this gene set were more surprising. For example, *ERG27,* although essential in both S. cerevisiae and *C. albicans,* lacked a clear growth phenotype in A. fumigatus. This phenotype cannot be attributed to the failure of the p*NiiA* promoter to sufficiently repress *ERG27* expression since we could construct a viable *ERG27* deletion mutant by gene disruption (Table 1). As *ERG27* defines the last “essential” step in the S. cerevisiae ergosterol biosynthetic pathway and ergosterol intermediates that accumulate in *ERG* mutants are implicated as causing the phenotypes/chemotypes resulting from disrupting specific steps in the pathway [28], one possibility is that the presumed ergosterol intermediate (possibly 4-methylzymosterol or 4,4-dimethylzymosterol) is not deleterious for A. fumigatus growth. Indeed, an extended p*NiiA*-CPR analysis of the ergosterol pathway reveals that additional genes defining the last essential steps in this pathway in S. cerevisiae (e.g., *ERG26*) are not essential in A. fumigatus (W. Hu, H. Wang, S. Sillaots, S. Kauffman, J. Becker, et al., unpublished data). Other interesting examples include the GDP-GTP exchange factors (GEF) *ROM2* and *CDC24* that regulate Rho1p and Cdc42p, respectively, in S. cerevisiae [29,30]. Again, *ROM2* was independently confirmed as nonessential in A. fumigatus as a viable knockout strain was constructed (Table 1) despite lacking a second clear homolog in A. fumigatus and sharing high homology (1e-155) to an essential *C. albicans* ortholog*.* In contrast, the A. fumigatus gene identified as sharing the greatest homology to *CDC24* lacks striking homology (7e-30) to S. cerevisiae or *C. albicans CDC24* and thus may be functionally distinct from *CDC24*. Regardless, it is unexpected that two GEF proteins critical to the process of polarized growth in yeast are either nonessential or absent from the A. fumigatus mycelial growth process, as is similarly the case for multiple polarized growth positional markers (e.g., *BUD3, BUD8,* and *BUD9*) [27]. Indeed, these examples stress the requirement for performing such analyses directly in A. fumigatus.

Our genetic analysis of the *A. fumigatus ERG11* gene family begins to address the relative role of each *ERG11* gene member during mycelial growth, as well as issues relating to differences in A. fumigatus activity among azole-based drugs*.* We demonstrate by genetic inactivation of either homolog that each enzyme can compensate for the lack of the other but that inactivation of both *ERG11A* and *ERG11B* is inviable in A. fumigatus. Therefore, azole-based antifungal drugs must inhibit both members of the *ERG11* gene family in order to display potent inhibitory activity against *A. fumigatus.* This may (in part) reflect the highly variable A. fumigatus MIC levels between azole-based drugs. In addition, we demonstrate that *ERG11A* mutants (rather than *ERG11B* mutants) are specifically hypersensitive to fluconazole, suggesting that *ERG11A* encodes the major 14α-demethylase activity contributing to the poor susceptibility of A. fumigatus to this drug. As *ERG11A* mutant also displays hypersensitivity to itraconazole and additional azoles [23], *ERG11A* may encode the major 14α-demethylase activity required for mycelial growth. Consistent with this view, multiple missense mutations of *ERG11A* (rather than *ERG11B*) have been linked to itraconazole and posoconazole resistance by UV mutagenesis studies and analysis of azole-resistant clinical isolates of A. fumigatus [31–33]. ERG11Ap may also possess an inherently greater resistance to azoles, although biochemical studies are required to address this possibility. Additional studies are also required to address the functional role of *ERG11B.* Although we demonstrate that it is not solely required for A. fumigatus pathogenesis, it is unclear whether *ERG11B* serves exclusively a redundant role with *ERG11A* or a more specialized function under particular conditions.

A variety of alternative strategies (see Introduction) to genetically investigate gene function in A. fumigatus are currently being pursued, and where possible, we have compared p*NiiA*-CPR terminal phenotypes to those reported [12–15]. Recently, a doxycycline-regulated gene expression system in A. fumigatus was reported*,* although it remains to be tested using an endogenous A. fumigatus gene [16]. Unlike previous reports, our work employs a directed strategy to systematically evaluate A. fumigatus essential genes on a relatively large scale and distinguish between cidal or static growth-arrested terminal phenotypes. Importantly, the regulatable promoter approach circumvents the need to complement mutants with their corresponding wild-type gene; a difficult and time-consuming process in A. fumigatus. The ability to recover multiple, independently generated, promoter replacement mutants by this method also serves to reinforce the gene-specific phenotypes observed. Unlike alternative strategies, we demonstrate that p*NiiA*-CPR mutants can be used to validate the gene essentiality in an immunocompromised murine model of infection. Pathogenesis studies assist prioritization of essential genes for drug screening and provide a genetic prediction that cognate inhibitors would display similar efficacy in an animal model of infection. Finally, unlike a genetic screen, this approach allows an up-front systematic examination of specific A. fumigatus genes that meet bioinformatic spectrum criteria and/or essential phenotypes in other fungal organisms and, hence, enables a rapid and focused survey of potential broad-spectrum antifungal drug targets. In this way, we demonstrate a significant enrichment in the identification and validation of essential genes in this pathogen and envision that its utility could be expanded*.*

The principal limitation to the p*NiiA*-CPR strategy is that, as with any other promoter replacement strategy, it will fail to correctly identify gene essentiality in cases where endogenous gene expression levels are naturally lower than the residual level achieved by repression of the *NiiA* promoter. Although our data demonstrate that such limitations are not common, we can neither rule out this complication nor predict its occurrence among individual genes tested. In cases where a more rigorous analysis of a single (or relatively small number of genes) is of particular interest, an independent verification of the null phenotype(s) should be performed by gene disruption, as performed in Table 1. An advantage of this approach is that the p*NiiA*-CPR strategy offers a means to identify the majority of essential genes in this pathogen. Minor technical limitations of this approach relate to the requirement to predict the gene's correct start codon, which can be particularly difficult for those A. fumigatus genes that contain extremely short 5′ exons. In addition, construction of p*NiiA*-CPR cassettes involves step-by-step subcloning to introduce sufficient flanking homology to each given gene. Greater throughput of cassette constructions could be achieved using gap repair methods amenable in S. cerevisiae [34]. Although relatively large regions of flanking homology are required to correctly target promoter replacement cassettes, shorter flanking regions could be used in conjunction with a recipient strain deleted of Ku70 or Ku80 [35,36].

Limitations to the murine model described also exist. Again, residual levels of expression of p*NiiA*-CPR mutants in vivo may not always be sufficient to produce an avirulent phenotype. This depends not only quantitatively on the differential level of repression achieved versus wild-type expression but also on the qualitative level of expression necessary to support function, which also can vary between genes and cannot be predicted. For example, we cannot rule out the possibility that the observed p*NiiA*-*AUR1* virulence (despite this gene displaying a 4+ growth phenotype) may reflect an insufficient shutoff within the host. Alternatively, the p*NiiA*-*AUR1* mutant may be suppressed in vivo by the uptake of host sphingolipids in a manner analogous to that reported of *Candida glabrata ERG11* and *ERG9* mutants (see below) [37,38]. Notwithstanding this limitation, multiple (*n* = 5) individual p*NiiA*-CPR mutants could unambiguously be confirmed as avirulent by this murine model, strongly suggesting its general utility (Figure 7B and 7C). Indeed, although potential drug targets may be missed, others are definitively identified, such as *GFA1,* which is directly confirmed as (1) essential for growth, (2) displaying a cidal terminal phenotype, and (3) avirulent in a murine model of pathogenesis. Although the murine model does not reflect a true pulmonary aspergillosis infection model (because mice were infected through tail vein injection rather than the normal route of infection, namely inhalation), such improvements can be envisioned. Also, unlike that of a tetracycline-regulatable promoter system [11], virulence of p*NiiA*-CPR mutants can only be evaluated from the perspective of prophylactic antifungal drug targets because repression is constitutive rather than controlled. Nonetheless, p*NiiA*-CPR mutants provide a unique advantage over alternative methods [12,13,15] to validate A. fumigatus genes in pathogenesis studies.

Formal demonstration that gene essentiality confirmed in vitro indeed confers an avirulent phenotype is necessary, as exemplified by phenotypic analysis of *ERG11* across fungal pathogens. Surprisingly, neither *ERG11* nor *ERG9* is essential in a murine model of C. glabrata infection, despite both genes being confirmed as essential in vitro [37,38]. Instead, *C. glabrata ERG11* and *ERG9* mutants are virulent in a host environment, and it is demonstrated that they scavenge host cholesterol to suppress ergosterol biosynthetic defects [37,38]. Direct experimentation was required to rule out that this phenotype extends to A. fumigatus. Although neither *ERG11A* nor *ERG11B* mutants were avirulent, the *ERG11* double mutant *(erg11B*Δ, p*NiiA*-*ERG11A)* is completely avirulent. In the case of novel antifungal targets, one can neither predict the consequences of their inactivation in an animal model nor neglect their direct experimental confirmation. Moreover, as A. fumigatus pathogenesis likely reflects its saprophytic lifestyle and virulence factors remain to be identified, a p*NiiA*-CPR strategy demonstrating avirulence of essential genes offers a broad set of potential drug targets.

In conclusion, the p*NiiA*-CPR strategy overcomes a number of historical challenges in applying molecular genetics to A. fumigatus and is highly amenable to genetically investigate A. fumigatus biology as well as to validate essential genes in this important human fungal pathogen. This approach facilitates their phenotypic analysis both in vitro and in vivo, and the resulting conditional mutants serve as target-based whole cell assays, thereby enabling the discovery, prioritization, and screening of novel antifungal targets directly in A. fumigatus. Indeed, promoter replacement strategies in C. albicans [11] and here in A. fumigatus encourage its broader application.

## Materials and Methods

### Strains, media, and cultural conditions.

A. fumigatus wild-type strain CEA10 and its uracil and uridine auxotroph, CEA17, were used in this study [39]. ACM and AMM were prepared as reported [40]. ACM supplemented with uracil and uridine was used to obtain CEA17 conidia suspensions and to produce mycelia for protoplast preparation. For protoplast transformation, a modified AMM was used that contained 10 mM Mg(NO_3_)_2_ as the sole nitrogen source and 1% (w/v) glucose as the sole carbon source (AMM plus nitrate). In addition, AMM (AMM plus ammonium) that contained 10 mM ammonium tartrate (C_4_H_12_N_2_O_6_; Sigma, http://www.sigmaaldrich.com) as the sole nitrogen source was used to repress the p*NiiA* for phenotypic analysis of p*NiiA*-CPR mutant. A. fumigatus was grown at 37 °C except where otherwise mentioned.

### Construction of plasmid pPyrG-p*NiiA.*

Plasmid pPyrG-p*NiiA* was constructed as follows (Figure 1A). Briefly, the *A. niger pyrG* selectable marker and the p*NiiA* sequence were amplified by PCR from plasmid pAB4-ARp [41] (kindly provided by Christophe d'Enfert, Institut Pasteur), and A. fumigatus genomic DNA, respectively, using gene-specific primers containing multiple cloning sites (Table S1). The above PCR products of *pyrG* and p*NiiA* were first digested with Not1/Nde1 and Nde1/Xho1, respectively, and then subcloned into pBluescript (SK; Stratagene, http://www.stratagene.com) at the Not1/Xho1 sites. The resulting plasmid is pPyrG-p*NiiA,* which contains *pyrG-*p*NiiA* cassette flanked on either side with a couple of cloning sites (Figure 1A).

### Construction of p*NiiA*-CPR cassette.

Through a subcloning strategy, CPR cassette was constructed that included the *A. niger PyrG* selectable marker and the p*NiiA* sequence flanked on either side with approximately 1.5 kb of gene-specific flanking sequence (Figure 1B). Briefly, the CPR cassette was constructed in two steps. In step 1, approximately 1.5-kb 5′-flanking sequence (left arm) that was usually 250 bp upstream of the ATG start codon of the target gene was amplified by PCR from A. fumigatus CEA10 genomic DNA with two gene-specific primers and then subcloned into suitable cloning sites (usually at Not1 and Mlu1) of the *pyrG* side of plasmid pPyrG-p*NiiA*. In step 2, approximately 1.5-kb downstream (3′-) genomic flanking sequence (right arm) that starts at the ATG start codon was amplified from A. fumigatus CEA10 genomic DNA with two gene-specific primers and then subcloned into the p*NiiA* side of plasmid pPyrG-p*NiiA* (usually at Asc1 and Pac1 sites). The resulting p*NiiA*-CPR cassette was excised from the plasmid by digesting with restriction enzymes at either end of the cassette (usually Not1 and Pac1) and used to transform A. fumigatus strain CEA17 (see below).

### Protoplast preparation and transformation.

Promoter replacement cassette (p*NiiA*-CPR cassette) DNA was introduced into A. fumigatus strain CEA17 *(pyrG^−^)* by protoplast transformation according to established procedures with minor modifications [42]. Briefly, protoplasts were prepared with freshly grown mycelium using lysing enzyme (Sigma) (0.5 g for 50 ml digestion). From 5 to 10 μg of excised CPR-cassette DNA was used for each transformation, and the transformants were grown at 37 °C for 3 d on AMM (AMM plus 10 mM nitrate) containing 1.2 M sorbitol as osmotic stabilizer but lacking uridine and uracil. Three days post-transformation, transformants were transferred to fresh AMM plus nitrate plates and then subjected to genotypic and phenotypic analysis.

### Genotypic analysis.

A PCR strategy was used to confirm double-crossover integrated promoter replacements in mutants in which the endogenous promoter of the target gene has been precisely replaced with p*NiiA* (Figure 1B). Genomic DNA was prepared using the DNeasy plant kit (Qiagen, http://www.qiagen.com) from transformants grown on AMM plus nitrate and then subjected to genotypic analysis by PCR. Three PCRs were performed, of which two mapped the promoter replacement junctions by using two sets of primers (L1 and L2 for left-arm junction, R1 and R2 for right-arm junction), with the third PCR using primer set L1/P to confirm the presence of the endogenous promoter in wild-type CEA10 but not in the PR mutants (Figure 1B). p*NiiA*-CPR mutants that have been confirmed by genotypic analysis were subjected to monoconidial purification, and the resulting monoconidial isolates were reconfirmed by PCR as described above.

### Phenotypic analysis of CPR mutants.

The phenotypes of all CPR mutants were evaluated by comparing the growth phenotype on inducing (AMM plus 10 mM nitrate) and repressing (AMM plus 10 mM ammonium) conditions. Typically, the terminal growth phenotype was observed after 36 to 40 h at 30 °C by streaking conidiospores onto inducing and repressing media. Qualitative scores were assigned to each of the CPR mutants to classify the severity of the terminal growth phenotype observed. A 4+ phenotype was assigned to CPR mutants that failed to grow in repressed media (AMM plus ammonium). Other growth phenotypes were scored as 3.5+ (very strong), 3+ (strong), 2+ (mild), 1+ (minor), or 0+ (no growth phenotype) (Figure 1C).

### Cidal/static phenotypic analysis.

To determine the terminal cidal/static phenotypes of A. fumigatus essential genes, 1 × 10^3^ to 1 × 10^4^ conidiospores were incubated in broth AMM plus 10 mM ammonium tartrate (repressing condition) for 0 (time zero control), 24, 48, and 72 h; washed with PBS twice; and then plated onto inducing media plate (AMM plus 10 mM nitrate) for CFU counting. A cidal phenotype was assigned to those genes whose conditional mutant displayed a significant CFU reduction (greater than 90%) after 24 h under repressing conditions. Slow cidal targets are those that displayed reduced cell viability after 48 or 72 h, respectively.

### Immunocompromised murine model of systemic infection.

A. fumigatus strains were tested for virulence in an immunocompromised murine model for systemic infection using CD-1 male mice (22 to 25 g; Charles River Laboratories, http://www.criver.com). Mice were housed five per cage with food and water supplied ad libitum. All experiments were performed according to the National Institutes of Health guidelines for the ethical treatment of animals. Mice were rendered immunocompromised by treatment with cyclophosphamide at 150 mg/kg 4 d and 1 d prior to infection and 100 mg/kg twice a week after infection. A. fumigatus conidial suspensions were prepared from 4- to 5-d-old cultures grown on solid medium by harvesting the conidia with gentle shaking by hand using 5 ml of PBS, 0.2% Tween-20, and 3 ml of glass beads. The conidial suspension was centrifuged (3,000*g,* 5 min), the pellet was resuspended in 0.01% Tween-20, and the conidia were counted on a hemacytometer. Immunocompromised mice were infected by tail vein injection of 0.1 ml of a conidial suspension of 1 × 10^6^ conidia/ml. After injection, the conidia suspensions were diluted and plated to determine the viable count of the inoculum. Mice were monitored three times daily for signs of infection, up to 12 to 22 d. Animals were considered moribund when they could no longer reach food or water. Moribund mice, along with those surviving to the end of the experiment, were killed by anesthetization followed by cervical dislocation. All tested CPR mutants were coded so that laboratory workers did not know the identity of the strains. For each strain, five mice were infected.

### Essential gene validation by ORF disruption.

To disrupt the complete ORF of a target gene, a disruption cassette was constructed. Briefly, 5′-flanking sequence (approximately 1.5 kb, upstream of the ATG start codon) of the target gene was amplified by PCR using two gene-specific primers and then subcloned into the Not1/Mlu1 sites of plasmid pPyrG-p*NiiA* (Figure 1A). Similarly, the 3′-flanking sequence (approximately 1.5 kb, downstream of the stop codon) was amplified by PCR and subsequently subcloned into the Nde1/Pac sites of plasmid pPyrG-p*NiiA*. The resulting plasmid was digested using Not1/Pac1 and then introduced into CEA17 strain by protoplast transformation (see above). Transformants were incubated on ACM without uracil and uridine and then subjected to genotypic analysis by PCR using primer sets to verify the disruption junctions and ORF disruption. Gene essentiality was inferred if no genotype-confirmed transformants could be recovered from a large population of transformants (*n* > 80). Otherwise, if genotype-confirmed transformants were recovered, the target gene was defined as nonessential.

### *ERG11* double knockout.

To validate the gene essentiality of the *ERG11A/ERG11B* gene family, a double knockout mutant was constructed as follows. In step 1, an *ERG11B* null mutant *(erg11B*Δ*)* was constructed by disruption of the whole *ERG11B* ORF using a *pyrG*-based gene disruption cassette as described above. In step 2, the *pyrG* selection marker was recycled from *erg11B*Δ to facilitate the promoter replacement of *ERG11A*. To achieve this, the *ERG11B* gene disruption plasmid was digested with Mlu1 and Asc1 to remove the *PyrG*-p*NiiA* insert from the cassette and religated (Mlu1 and Asc1 provide compatible 5′ overhangs) prior to subsequent transformation with Escherichia coli. The resulting plasmid was then purified and digested with Not1 and Pac1 to excise the *ERG11B* insert (comprising fused 5′ and 3′ flanking sequences and no intervening *pyrG*-p*NiiA* insert) and used to transform *erg11B*Δ. Transformants were grown at 30 °C for 48 h on ACM containing 5 mM uridine and uricine, respectively. The conidiospores of resulting transformants were collected and then plated onto AMM plates containing 10 mM nitrate and 1 mg/ml 5-FOA to counterselect *pyrG* ^−^ strains. The resulting *pyrG* ^−^ strain was then genotyped by PCR to confirm that the *pyrG* ^−^ selectable marker has been removed from *erg11B*Δ. In step 3, the resulting *pyrG* ^−^ *, erg11B*Δ strain was then used as the starting strain to perform promoter replacement of *ERG11A* as described above.

### Construction of *TUB1* tandem duplicate mutant.

To construct *TUB1* tandem duplicate mutants, *TUB1* ORF plus approximately 1 kb of the DNA sequence directly downstream of the stop codon was amplified by PCR and then subcloned into the Asc1 and Pac1 sites of plasmid pPyrG-p*NiiA* (Figure 1A). The resulting plasmid was used to transform A. fumigatus CEA17 by protoplast transformation as described above. Transformants were incubated on AMM plus nitrate without uracil and uridine and then subjected to genotypic analysis by PCR using primer sets to verify single crossover event.

### RT-PCR and real-time RT-PCR.

To perform RT-PCR, p*NiiA*-CPR mutants and wild-type were grown either in AMM plus nitrate (inducing) or in AMM plus ammonium (repressing) at 37 °C for 20 h. Total RNA was extracted using the RNeasy plant mini kit (Qiagen) according to the manufacturer's instructions. Total RNA samples were treated with Turbo-DNase I at 37 °C for 30 to 40 min according to the manufacturer's manual (Ambion, http://www.ambion.com). RNA samples obtained using these procedures lacked detectable genomic DNA as assayed by control PCR tests. The first-strand cDNA was synthesized by using ThermoScript RT-PCR system and oligo(dT) primers (Invitrogen, http://www.invitrogen.com) according to the manufacturer's instructions. Double-strand cDNAs were then amplified from the above first-strand cDNA by PCR using a set of gene-specific forward and reverse primers (Table S1). A typical PCR program is as the following: 94 °C for 2 min, 1 cycle; 94 °C for 30 s, 60 °C for 30 s and 72 °C for 2 min, 16 to 20 cycles; and then extension at 72 °C for 10 min. PCR products were then subject to 1% agarose-gel analysis.

To perform real-time RT-PCR, total RNA samples was extracted and treated with Turbo-DNase (Ambion), and the first-strand cDNA was synthesized as described above. Real-time PCR was then performed using the ABI 7500 thermocycler (Applied Biosystems, http://www.appliedbiosystems.com). Reactions containing cDNA, forward and reverse primers, and a fluorescent probe (integrated DNA technologies, invasive aspergillosis, and applied biosystems; see Table S2) and TaqMan Master Mix were run in triplicate using the standard conditions suggested by the manufacturer. When possible, primers and probes were specifically designed to cross adjacent exons to avoid amplification of trace genomic DNA. The mean threshold cycle value (C_t_) for each sample was used to calculate cDNA abundance under inducing or repressing growth conditions, and samples were normalized based on total input RNA as previously described [43]. Changes in expression were calculated relative to the C_t_ for each gene expressed by a wild-type strain.

### Northern blot analysis.

Northern blot analysis was carried out according to established procedures [14,15]. Briefly, total fungal RNA was prepared using the RNeasy plant mini kit (Qiagen) according to the manufacturer's instructions. From 5 to 10 μg of total RNA was used for each sample. P^32^-labeled *ALR1* and *ACT1* probes were made from PCR products amplified using gene-specific primers (Table S1).

### Drug susceptibility testing.

MIC testing of antifungal drugs against A. fumigatus wild-type strain CEA10 and conditional mutants constructed from promoter replacement was performed by broth microdilution as described by the National Committee for Clinical Laboratory Standards with minor modifications [44]. The A. fumigatus inoculum was adjusted to concentrations of approximately 2 × 10^3^ conidiospores/ml in AMM, and an aliquot of 0.1 ml was added to microtiter wells containing 0.1 ml of antifungal drug solutions in AMM. For inducing conditions, AMM plus 10 mM Mg(NO_3_)_2_ was used. To sensitize the CPR mutants, ammonium tartrate was added to AMM plus 10 mM nitrate at a final concentration of 10 to 160 μM. The microtiter plate was incubated at 37 °C for 48 h, and the MICs were determined as the lowest concentration that completely inhibited mycelia growth.

## Supporting Information

Figure S1 Microscopic Analysis of p*NiiA*-*nudC* Terminal phenotypes (A) Phase contrast microscopy (×160) demonstrates normal hyphal growth of p*NiiA*-*nudC* mutants incubated under inducing conditions (16 h at 30 °C).
(B) Phase contrast microscopy demonstrates p*NiiA*-*nudC* conidia typically failed to germinate (greater than 70%) and a minority (less than 30%) formed short growth-arrested germ tubes when incubated under repressing conditions (16 h at 30 °C).
(C and D) Higher magnification (×400) phase contrast microscopy and DAPI staining of p*NiiA*-*nudC* mutant incubated under the same conditions as above.
(E and F) DAPI staining demonstrates a nuclear distribution phenotype of the p*NiiA*-*nudC* mutant as multiple nuclei remain inside the ungerminated conidia and short growth-arrested germ tubes under repressing conditions (F) but not in mycelia under inducing conditions (E).
(5.3 MB PPT)

Figure S2 RT-PCR Analysis of Expression Level of Multiple p*NiiA*-CPR Mutants p*NiiA*-CPR mutants were grown in AMM plus nitrate (In) or AMM plus ammonium (Re) at 37 °C for 20 h, and total RNA was extracted and normalized. To monitor and ensure even sample loading, RT-PCRs for the *ACT1* transcript were also performed using identical samples (unpublished data). Standard PCR was also performed to confirm that there is no detectable genomic-DNA contamination (unpublished data).
(197 KB PPT)

Figure S3 Normal Hyphal Growth of p*NiiA*-CPR Mutants under Inducing Conditions To compare terminal phenotypes characterized in Figure 4, p*NiiA*-CPR mutants of *GFA1, TUB1, SEC31, SLY1, ERG10, HEM15, PRI1,* and *FKS1* were grown on AMM plus nitrate (inducing conditions) at 30 °C for 2 d. Micrographs were taken under a microscope at a magnification of approximately ×80.
(299 KB PPT)

Table S1 Primers Used for p*NiiA* Cassette Construction, Northern Blot, and RT-PCR (35 KB DOC)

Table S2 Forward and Reverse Primers and Probes Used in Real-Time RT-PCR (26 KB DOC)

## Abbreviations

ACM: Aspergillus complete medium
AMM: Aspergillus minimal medium
CFU: colony-forming units
CPR: conditional promoter replacement
MIC: minimum inhibitory concentration
*pNiiA*: A. fumigatus *NiiA* nitrogen-regulatable promoter
RT-PCR: reverse transcription PCR
