# Supplementary material for: Essential Gene Identification and Drug Target Prioritization in Aspergillus fumigatus
Source: PLoS Pathog. 2007 Mar 9;3(3):e24. doi: 10.1371/journal.ppat.0030024 (PMC1817658; doi:10.1371/journal.ppat.0030024)
Supplement: Supplementary file 1 [file ppat.0030024.st001.doc]

**Supplementary Table S1. Primers used for pNiiA cassette construction, Northern blot and RT-PCR**

| Gene | Forward primer (5'-3') | Reverse primer (5'-3') | Note |
| --- | --- | --- | --- |
| *ACT1* | TTGCTGCTCT CGTCATCGACAAT | GAAACGCTCG TTACCAATGGTG | To amplify *ACT1* probe |
| *ACT1* | TTGCTGCTCT CGTCATCGACAAT | GAAACGCTCG TTACCAATGGTG | *ACT1* RT-PCR |
| *ALR1* | GAATCGAAGCTACCGCTGAT | GTTCGCATAGACCTTATCGG | To amplify *ALR1* probe |
| *ALR1* | CTCGAATCGAAGCTACCGCTGAT | CGCATGCACAGTGCTCTGGGACT | *ALR1* RT-PCR |
| *ERG11A* | TGTCCCAATTCCAAGCTGATGGA | TGTCCCAATTCCAAGCTGATGGA | *ERG11A* RT-PCR |
| *ERG11B* | GTCGACATGTTGTGTATGACTGCCCT | TCAGGCTTTGGTAGCGGACTCG | *ERG11B* RT-PCR |
| *ERG27* | CCAAGGCAATCTGGGGCGTTAT | TCATGCCTCGGATTTTGCCTGGG | *ERG27* RT-PCR |
| *MET2* | AGT TGCCTCCTTGCCTGAAAATC | TACCACGCAG TGATATCCTCGAC | *MET2* RT-PCR |
| *niiA/niiD* promoter | GGAATTCCATATGTGCTCAGAGTACTACAG CTGATAAT | CCGCTCGAGCGGTTAATTAAGGCGGCTAGCATGGCGCGCCCGTGACGAAGTCTCAACGCCAGT | To amplify *A. fumigatus* *niia/niaD* promoter (p*NiiA*) |
| *pyrG* | ATAAGAATGCGGCCGCACATGTCATGCATGCCGACGCGTGTTTAAACTCCTCCTGGTAGCTGGACTGAAG | GGAATTCCATATGCGATCGAGCATCCAACTAAACTAG | To amplify *A. niger pyrG* |
| *ROM2* | CAAGGGTGGTTACGAGCAGACCC | TGTTGTTGGGGCTTGTTCCAGAA | *ROM2* RT-PCR |
