# Supplementary material for: Essential Gene Identification and Drug Target Prioritization in Aspergillus fumigatus
Source: PLoS Pathog. 2007 Mar 9;3(3):e24. doi: 10.1371/journal.ppat.0030024 (PMC1817658; doi:10.1371/journal.ppat.0030024)
Supplement: Supplementary file 2 [file ppat.0030024.st002.doc]

**Supplementary Table S2. Forward and reverse primers and probes used in real-time RT-PCR.**

| **Gene** | **Forward primer** (5'-3') | **Probe**^*^ (5'-3') | **Reverse primer** (5'-3') |
| --- | --- | --- | --- |
| *ALR1* | GTGATACTTTCCGGGATCTCTTTCA | CCTCCCTCCGGGCCAA | GTACGTCCAACCACCACACA |
| *MET2* | GGCCATTCATAATGATGGTCACAAG | CCGGCTCTCCCGTCAGT | GCCGCTTCCTGTCACTGTT |
| *TUB1* | CCAACTCTTGCTGGGAGCTCTA | TGCTTGGAGCACGGC | TCAAGTAACCATCGGGCTGAA |

^*^ All probes are 5’FAM-labeled
